# Health Care Resource Utilization in Management of Opioid-Naive Patients With Newly Diagnosed Neck Pain

**DOI:** 10.1001/jamanetworkopen.2022.22062

**Published:** 2022-07-13

**Authors:** Michael C. Jin, Michael Jensen, Zeyi Zhou, Adrian Rodrigues, Alexander Ren, Maria Isabel Barros Guinle, Anand Veeravagu, Corinna C. Zygourakis, Atman M. Desai, John K. Ratliff

**Affiliations:** 1 Department of Neurosurgery, Stanford University School of Medicine, Stanford, California

## Abstract

**Importance:**

Research has uncovered heterogeneity and inefficiencies in the management of idiopathic low back pain, but few studies have examined longitudinal care patterns following newly diagnosed neck pain.

**Objective:**

To understand health care utilization in patients with new-onset idiopathic neck pain.

**Design, Setting, and Participants:**

This cross-sectional study used nationally sourced longitudinal data from the IBM Watson Health MarketScan claims database (2007-2016). Participants included adult patients with newly diagnosed neck pain, no recent opioid use, and at least 1 year of continuous postdiagnosis follow-up. Exclusion criteria included prior or concomitant diagnosis of traumatic cervical disc dislocation, vertebral fractures, myelopathy, and/or cancer. Only patients with at least 1 year of prediagnosis lookback were included. Data analysis was performed from January 2021 to January 2022.

**Main Outcomes and Measures:**

The primary outcome of interest was 1-year postdiagnosis health care expenditures, including costs, opioid use, and health care service utilization. Early services were those received within 30 days of diagnosis. Multivariable regression models and regression-adjusted statistics were used.

**Results:**

In total, 679 030 patients (310 665 men [45.6%]) met the inclusion criteria, of whom 7858 (1.2%) underwent surgery within 1 year of diagnosis. The mean (SD) age was 44.62 (14.87) years among nonsurgical patients and 49.69 (9.53) years among surgical patients. Adjusting for demographics and comorbidities, 1-year regression-adjusted health care costs were $24 267.55 per surgical patient and $515.69 per nonsurgical patient. Across all health care services, $95 379 949 was accounted for by nonsurgical patients undergoing early imaging who did not receive any additional conservative therapy or epidural steroid injections, for a mean (SD) of $477.53 ($1375.60) per patient and median (IQR) of $120.60 ($20.70-$452.37) per patient. On average, patients not undergoing surgery, physical therapy, chiropractic manipulative therapy, or epidural steroid injection, who underwent either early advanced imaging (magnetic resonance imaging or computed tomography) or both early advanced and radiographic imaging, accumulated significantly elevated health care costs ($850.69 and $1181.67, respectively). Early conservative therapy was independently associated with 24.8% (95% CI, 23.5%-26.2%) lower health care costs.

**Conclusions and Relevance:**

In this cross-sectional study, early imaging without subsequent intervention was associated with significantly increased health care spending among patients with newly diagnosed idiopathic neck pain. Early conservative therapy was associated with lower costs, even with increased frequency of therapeutic services, and may have reduced long-term care inefficiency.

## Key Points

**Question:**

What are common health care utilization patterns for management of newly diagnosed acute neck pain and how are they associated with care delivery?

**Findings:**

In this cross-sectional study of 679 030 adults conducted between 2008 and 2015, early imaging was common and often occurred before conservative treatment. However, early conservative therapy before imaging was associated with lower health care costs and reduced opioid use.

**Meaning:**

These findings suggest a need for improved care standardization with prompt conservative therapy initiation to reduce treatment cost and increase effectiveness.

## Teaser

This cross-sectional study examines health care utilization in patients with new-onset idiopathic neck pain.

## Introduction

Health care expenditures in 2016 were approximately 17.8% of the US gross domestic product.^1^ US expenditures for low back and neck pain accounted for approximately $87.6 billion in 2013, with spending increasing by $57.2 billion since 1996.^2^ Previous investigations^3,4,5^ have described health care resources for new-onset low back pain, but expenditure of resources in management of idiopathic neck pain is not well understood.

The source of neck pain is multifocal and may result from degeneration of the uncovertebral and/or zygapophyseal joints, disc herniation, trauma, or myofascial pain.^6,7^ Rarer causes include tumor, infection, and inflammation. In patients presenting with new-onset neck pain, clinicians must obtain a detailed examination and history to rule out any alarming symptoms (eg, fever, myelopathy, worsening neurological deficit, or incontinence) that would warrant emergent diagnostic studies and possible intervention.^8,9,10^ For patients with neck pain or upper extremity pain without alarming symptoms, the natural history, treatment approach, and potential need for operative management are poorly defined.^11,12,13,14^

Additional research is necessary to support care guidelines for treatment of new-onset neck pain; various clinical studies^15,16,17,18^ recommend conservative treatment modalities at initial presentation. These treatment regimens include physical therapy (PT), nonsteroidal anti-inflammatory drugs, epidural steroid injections (ESIs), opioids, traction, and bracing. Most acute symptoms subside within weeks with conservative treatment. If symptoms persist beyond 6 to 12 weeks, advanced imaging (eg, computed tomography or magnetic resonance imaging [MRI]) may be considered.^19,20,21^ However, we suspect high variability in patient management strategies may exist given the lack of consensus care guidelines. To understand care delivery efficiency, this cross-sectional study describes health care utilization and timing in opioid-naive patients with idiopathic neck pain.

## Methods

### Cohort and Study Design

Data used in this study were derived from the IBM Watson Health MarketScan Database, a nationally sourced administrative claims database spanning 2007 to 2016 and encompassing more than 75 million enrollees covered by eligible health care plans that has been used to explore diverse spinal pathologies.^22,23,24^ This study was approved by the Stanford University School of Medicine institutional review board and was conducted in accordance with Strengthening the Reporting of Observational Studies in Epidemiology (STROBE) reporting guidelines. Informed consent was not needed because the data were anonymous and publicly available, in accordance with 45 CFR §46.

All adult patients with newly diagnosed idiopathic cervical neck pain and no evidence of opioid use during the year preceding the index diagnosis date were included in our study. The index diagnosis date was denoted as the first date with a qualifying service or hospital admission indicating neck or upper extremity pain as indicated by *International Classification of Diseases, Ninth Revision,* coding (eTable 1 in the Supplement). Continuous follow-up extending from 1 year before the index diagnosis date to 1 year after the index diagnosis date was required for study inclusion, allowing confirmation of no prior neck pain diagnosis, no excluding diagnoses, and canvassing of comorbidities. Patients with known bone fractures, myelopathy, and/or cancer diagnoses were excluded. Those with a documented opioid prescription during the year before the index diagnosis date were excluded to differentiate acute neck pain from chronic pain syndromes. Comorbidities were included on the basis of the Elixhauser comorbidity index.^25^

Given the follow-up requirement, included patients received their initial diagnosis between 2008 and 2015. The primary outcome of interest was postindex health care costs, whereas the secondary outcome of interest was opioid use. Treatment for neck pain was defined as either PT, chiropractic manipulative therapy (CMT), ESIs, or surgery. Conservative therapy was defined as either PT or CMT. Unless explicitly noted, PT and CMT reflect categorization of services provided as defined by *Current Procedural Terminology* coding. Early services (either treatments, prescriptions, or imaging) were defined as those occurring within 30 days of the initial index diagnosis. Long-term opioid use was defined according to 2 criteria: either 180 prescribed-days^26^ or 6 prescriptions^27^ within 1 year of the index diagnosis date. The diagnosing practitioner was defined as the practitioner submitting the medical claim with an acute neck pain diagnosis on the index diagnosis date; from this claim, the submitting practitioner’s specialty was directly extracted.

Costs were defined as the total payment for all inpatient and outpatient services documented as medical claims after applying pricing guidelines such as fee schedules but before adjusting for deductibles and copayments. These were aggregated across all claims submitted during the year following the index diagnosis date. Opioid use was assessed on the basis of documented prescriptions identified as opioid receptor agonists by the American Health Formulary Service and categorized as Schedule II and III substances by the US Drug Enforcement Administration. Prescribed durations were directly extracted from the pharmaceutical claim. Only patients with plans covering outpatient pharmaceuticals were included.

### Statistical Analysis

Data analysis was performed from January 2021 to January 2022. Group comparisons of continuous variables were performed using either the Mann-Whitney *U *test or *t* test, and comparisons of categorical variables were performed using the χ^2^ test of independence. Cohort matching was performed using coarsened exact matching, using a *k-*to-*k* unweighted approach,^28,29^ generating matched cohorts stratified by receipt of cervical spine surgery using covariates described in Table 1. For continuous features included in coarsened exact matching, coarsening bins were established according to the Sturge rule, *K = *(1* + *log_2_*N*), where *N* indicates the number of observations and *K* indicates the number of bins. Regression-adjusted estimates were generated from a 2-part mixed-effects generalized linear model, *E*(Costs*|X*_1_*…X_n_*)* = Pr*(Costs* > *0,*X*_1_*…X_n_*) × *E*(Costs*|*Costs* > *0,*X*_1_*…X_n_*)* *×* Pr*(Costs* > *0*|X*_1_*…X_n_*), which reflects the probability of any health care spending modeled using a binomial logistic regression, whereas *E*(Cost*|*Costs* > *0,*X*_1_*…X_n_*) indicates the estimated aggregate health care costs modeled using a γ generalized linear model with a log-link; *E*(Cost) denotes expected costs, *X*_1_*…X_n_* denotes the number of included features, and *Pr*(Cost) denotes the probability of nonzero costs. Random effects were modeled at the state level to account for state-specific variations in legislation and practice patterns. Alternatively, multivariable linear regression was also used to assess contributions of diverse features to health care costs. Regression-adjusted marginal means were derived to assess the association of individual outliers with group estimates.^30^ Significance was set at 2-sided *P* < .05. Statistical analyses and graphical representations were performed using R statistical software version 4.0.0 (R Project for Statistical Computing) and GraphPad Prism statistical software version 8 (GraphPad Software). R packages used include *cem*, *survey*, *lme4*, and *emmeans*.

**Table 1.** Cohort Characteristics

Characteristic	Patients, No. (%)					
	Matched			Not matched		
	Nonsurgical (n = 7607)	Surgical (n = 7607)	*P* value	Nonsurgical (n = 671 172)	Surgical (n = 7858)	*P* value
Age group, y						
18-29	110 (1.4)	110 (1.4)	>.99	113 863 (17.0)	104 (1.4)	<.001
30-39	1019 (13.4)	1019 (13.4)		139 510 (20.8)	1029 (13.1)	
40-49	2791 (36.8)	2791 (36.8)		170 968 (25.5)	2866 (36.5)	
50-59	2529 (33.2)	2529 (33.2)		144 851 (21.6)	2612 (33.2)	
60-69	1012 (13.3)	1012 (13.3)		68 565 (10.2)	1080 (13.7)	
70-79	124 (1.6)	124 (1.6)		18 496 (2.8)	141 (1.8)	
≥80	22 (0.3)	22 (0.3)		14 919 (2.2)	26 (0.3)	
Age, mean (SD), y	49.49 (9.53)	49.49 (9.53)	.99	44.62 (14.87)	49.69 (9.53)	<.001
Sex						
Male	4505 (59.2)	4505 (59.2)	>.99	306 016 (45.6)	4649 (59.2)	<.001
Female	3102 (40.8)	3102 (40.8)		365 156 (54.4)	3209 (40.8)	
Comorbidities						
Congestive heart failure	29 (0.4)	29 (0.4)	>.99	6654 (1.0)	53 (0.7)	.006
Cardiac arrhythmia	182 (2.4)	182 (2.4)	>.99	23 417 (3.5)	229 (2.9)	.006
Valvular disease	103 (1.4)	103 (1.4)	>.99	13 767 (2.1)	150 (1.9)	.40
Pulmonary circulation disorders	14 (0.2)	14 (0.2)	>.99	2007 (0.3)	30 (0.4)	.22
Peripheral vascular disorders	68 (0.9)	68 (0.9)	>.99	10 169 (1.5)	102 (1.3)	.13
Hypertension, uncomplicated	2026 (26.6)	2026 (26.6)	>.99	134 400 (20.0)	2183 (27.8)	<.001
Hypertension, complicated	64 (0.8)	64 (0.8)	>.99	8082 (1.2)	100 (1.3)	.62
Paralysis	<10 (NA)	<10 (NA)^a^	>.99	523 (0.1)	17 (0.2)	<.001
Other neurological disorders	94 (1.2)	94 (1.2)	>.99	12 295 (1.8)	123 (1.6)	.09
Chronic pulmonary disease	485 (6.4)	485 (6.4)	>.99	43 996 (6.6)	555 (7.1)	.07
Diabetes, uncomplicated	780 (10.3)	780 (10.3)	>.99	51 084 (7.6)	883 (11.2)	<.001
Diabetes, complicated	134 (1.8)	134 (1.8)	>.99	10 917 (1.6)	188 (2.4)	<.001
Hypothyroidism	458 (6.0)	458 (6.0)	>.99	45 392 (6.8)	512 (6.5)	.40
Kidney failure	25 (0.3)	25 (0.3)	>.99	5613 (0.8)	44 (0.6)	.009
Liver disease	89 (1.2)	89 (1.2)	>.99	8303 (1.2)	119 (1.5)	.03
Peptic ulcer disease excluding bleeding	19 (0.2)	19 (0.2)	>.99	1401 (0.2)	31 (0.4)	.001
AIDS/HIV	11 (0.1)	11 (0.1)	>.99	920 (0.1)	15 (0.2)	.26
Rheumatoid arthritis or collagen disorder	100 (1.3)	100 (1.3)	>.99	10 800 (1.6)	134 (0.0)	.53
Coagulopathy	<10 (NA)^a^	<10 (NA)^a^	>.99	2560 (0.4)	26 (0.3)	.53
Obesity	250 (3.3)	250 (3.3)	>.99	24 874 (3.7)	299 (3.8)	.67
Weight loss	39 (0.5)	39 (0.5)	>.99	4845 (0.7)	61 (0.8)	.62
Fluid and electrolyte disorders	88 (1.2)	88 (1.2)	>.99	11 238 (1.7)	122 (1.6)	.43
Blood loss anemia	<10 (NA)^a^	<10 (NA)^a^	>.99	1094 (0.2)	<10 (NA)^a^	.04
Deficiency anemia	57 (0.7)	57 (0.7)	>.99	10 195 (1.5)	91 (1.2)	.01
Alcohol abuse	40 (0.5)	40 (0.5)	>.99	4650 (0.7)	58 (0.7)	.68
Drug abuse	22 (0.3)	22 (0.3)	>.99	3360 (0.5)	34 (0.4)	.44
Psychoses	<10 (NA)^a^	<10 (NA)^a^	>.99	2948 (0.4)	22 (0.3)	.04
Depression	526 (6.9)	526 (6.9)	>.99	52 936 (7.9)	598 (7.6)	.38

Abbreviation: NA, not applicable.

^a^ Percentages could not be calculated for cells of fewer than 10 participants.

## Results

### Cohort Characteristics

In total, 679 030 patients (310 665 men [45.6%]) were included in our cohort (eFigure 1 in the Supplement). Of these, 7858 (1.2%) eventually underwent surgery during the year following the index diagnosis. Most of the cohort was younger than 65 years (630 273 patients [92.8%]). The mean (SD) age was 44.62 (14.87) years among nonsurgical patients and 49.69 (9.53) years among surgical patients. Surgical patients were generally older, frequently male, and had higher comorbidity burden compared with nonsurgical patients (Table 1). Among nonsurgical patients, 183 330 (27.3%) received PT, 149 807 (22.3%) received CMT, and 11 690 (1.7%) received ESIs during the year after diagnosis. Among surgical patients, 3150 (40.1%) received PT, 555 (7.1%) received CMT, and 1600 (20.4%) received ESIs. Among nonsurgical patients, 281 497 (41.9%) underwent early imaging within 30 days of the index diagnosis date. More specifically, 188 268 patients (28.1%) underwent radiography, 71 360 (10.6%) underwent advanced imaging, and 21 869 (3.3%) underwent both radiography and advanced imaging. A minority of patients pursued early PT (156 608 nonsurgical patients [23.3%] and 1482 surgical patients [18.9%]). Few patients received ESI within 1 month of the index diagnosis (4088 nonsurgical patients [0.61%] and 652 surgical patients [8.3%]). Among patients who received no treatment (surgery, PT, CMT, or ESI) for neck pain during the year following the index diagnosis, 199 737 patients (46.2%) underwent early imaging (126 973 patients [29.4%] underwent radiography only, 59 673 patients [13.8%] underwent advanced imaging only, and 13 091 patients [3.0%] underwent both).

### Long-term Costs

During the year after the index diagnosis, nonsurgical care for patients with neck pain accounted for $346 069 711 ($515.62 per patient) and surgical care accounted for $189 826 915 ($24 157.15 per patient) in total health care expenditures. After matching patients according to demographics and comorbidities, regression-adjusted estimates of per capita health care spending were $24 267.55 per surgical patient and $515.69 per nonsurgical patient (Table 2). Compared with 600 304 nonsurgical patients (89.4%), only 3926 surgical patients (50.0%) incurred no health care costs between months 6 and 12 postdiagnosis.

**Table 2.** Per Capita Estimation of Health Care Costs and Opioid Use (1 Year After Diagnosis)

Characteristics	Estimate, mean (SD)					
	Matched			Not matched		
	Nonsurgical	Surgical	*P* value	Nonsurgical	Surgical	*P* value
Health care costs, $						
Unadjusted	557.05 (1516.83)	24 240.83 (20 716.16)	<.001	515.62 (1578.86)	24 157.15 (21 091.8)	<.001
Regression adjusted	553.39 (124.88)	24 267.55 (4658.68)	<.001	515.69 (93.17)	24 327.57 (4044.37)	<.001
Opioid use, No. of prescribed-days						
Unmatched	5.87 (27.12)	32.26 (52.77)	<.001	5.63 (27.43)	32.51 (53.03)	<.001
Regression adjusted	5.64 (1.78)	32.52 (7.93)	<.001	5.63 (2.61)	33.66 (9.62)	<.001

Among nonsurgical patients, imaging constituted 26.0%, PT constituted 24.2%, CMT constituted 11.7%, and ESI constituted 3.8% of total costs. Among patients not receiving any treatments for neck pain (surgery, PT, CMT, or ESI), imaging constituted 36.6% of total health care spending. In total, patients undergoing early imaging but no additional treatments (surgery, PT, CMT, or ESI) accumulated $95 379 949 in health care costs during the year after the index diagnosis (199 737 nonsurgical patients [70.2%]; mean [SD], $477.53 [$1375.60] per patient; median [IQR], $120.60 [$20.70-$452.37] per patient), constituting 27.6% and 17.8% of the costs accumulated by the nonsurgical and total cohorts, respectively. Among patients who received neither surgery, PT, CMT, nor ESI, those who underwent early imaging accumulated significantly higher health care costs during the year after the index diagnosis (median [IQR], $120.60 [$20.70-$452.37] per patient vs $76.31 [$29.52-$180.64] per patient; nonparametric *P* < .001). This difference was marked in patients undergoing either only early advanced imaging or both early radiographic and early advanced imaging, who averaged $850.69 and $1181.67 per patient, respectively, during the year after the index diagnosis.

**Figure 1.**
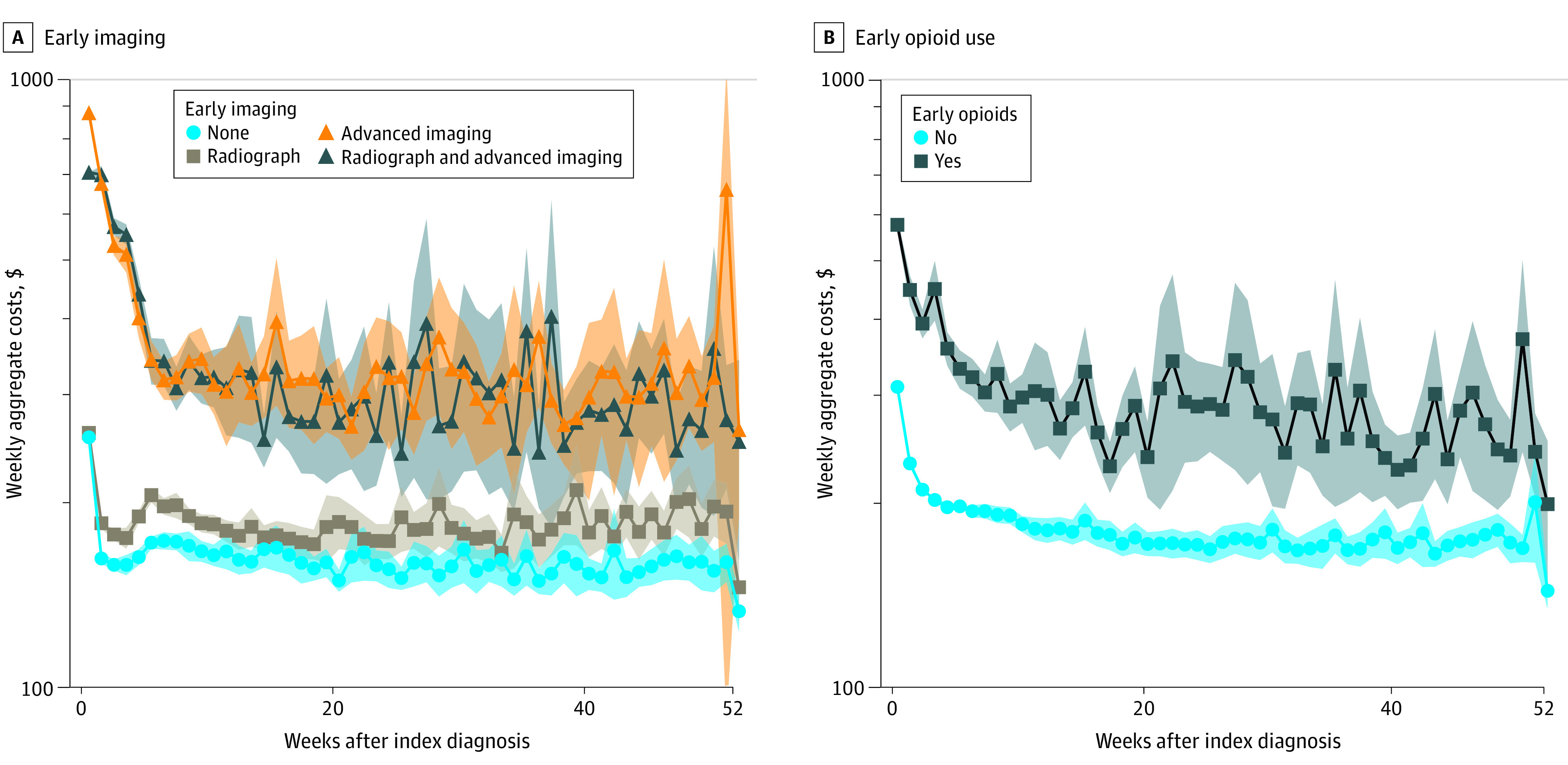
Weekly Health Care Utilization Following Early Imaging and Opioids Graphs show that early imaging (A) and early opioid use (B) were associated with elevated health care utilization in terms of weekly aggregate costs. Shaded areas denote 95% CIs.

**Figure 2.**
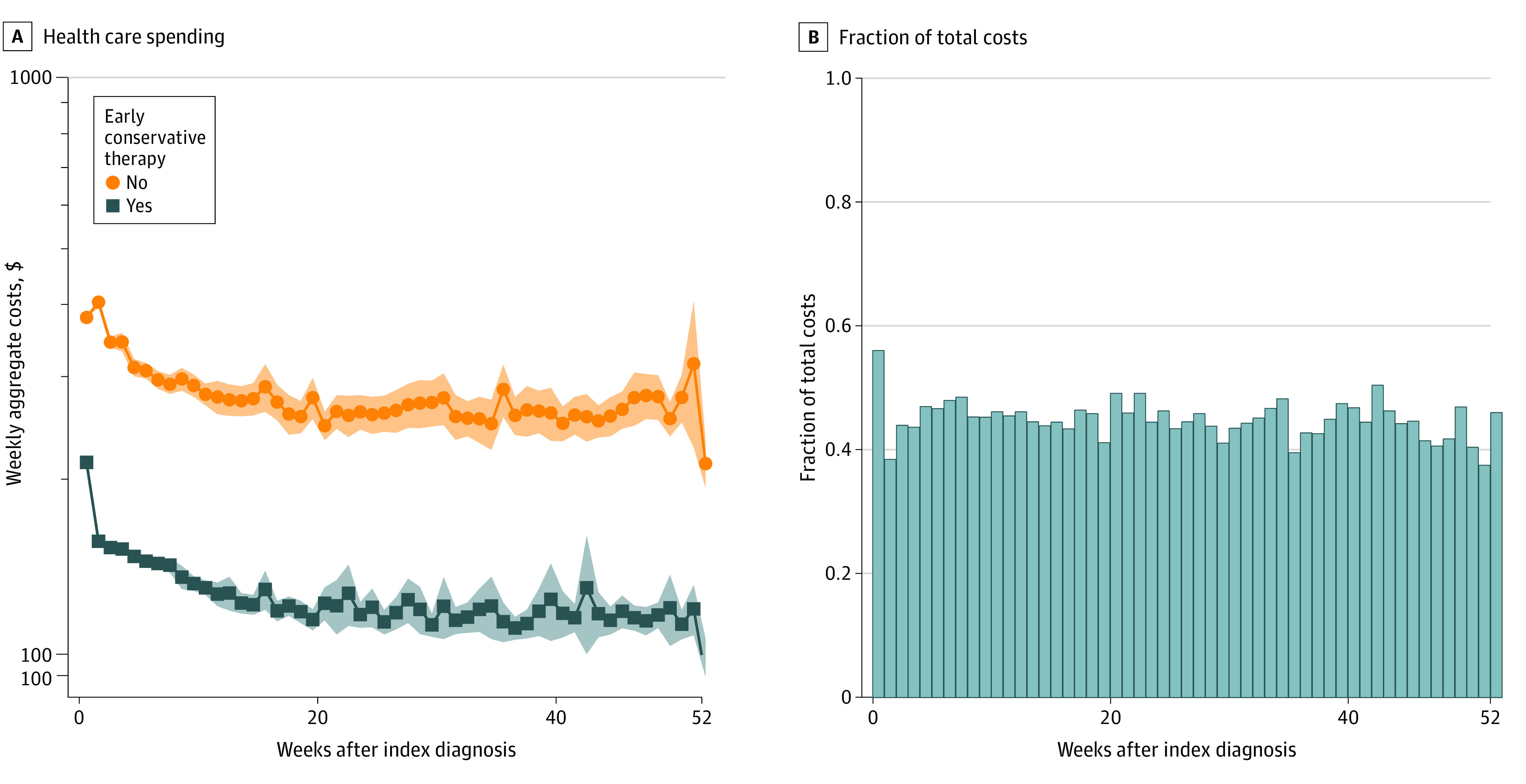
Weekly Health Care Utilization Following Early Conservative Therapy Graphs show that early conservative therapy use was associated with decreased long-term health care spending (A), resulting in 40% to 65% decreased costs (B). Shaded areas indicate 95% CIs.

Weekly health care costs were aggregated for nonsurgical patients stratified by use of early imaging, early opioids, and early conservative therapy. Use of early imaging was associated with significantly higher weekly costs, particularly among patients undergoing early advanced imaging (Figure 1A). Early opioid use was also associated with significantly higher weekly long-term health care costs (Figure 1B). Conversely, early use of conservative therapy (either CMT or PT) was associated with significantly decreased weekly health care costs (Figure 2A), ranging from 35% to 60% of average costs among patients not pursing early conservative therapy (Figure 2B). After adjusting for demographics, comorbidities, and surgery, use of early conservative therapy remained associated with 24.8% (95% CI, 23.5%-26.2%) lower long-term health care costs (eTable 2 in the Supplement).

### ESIs and Opioid Use

**Figure 3.**
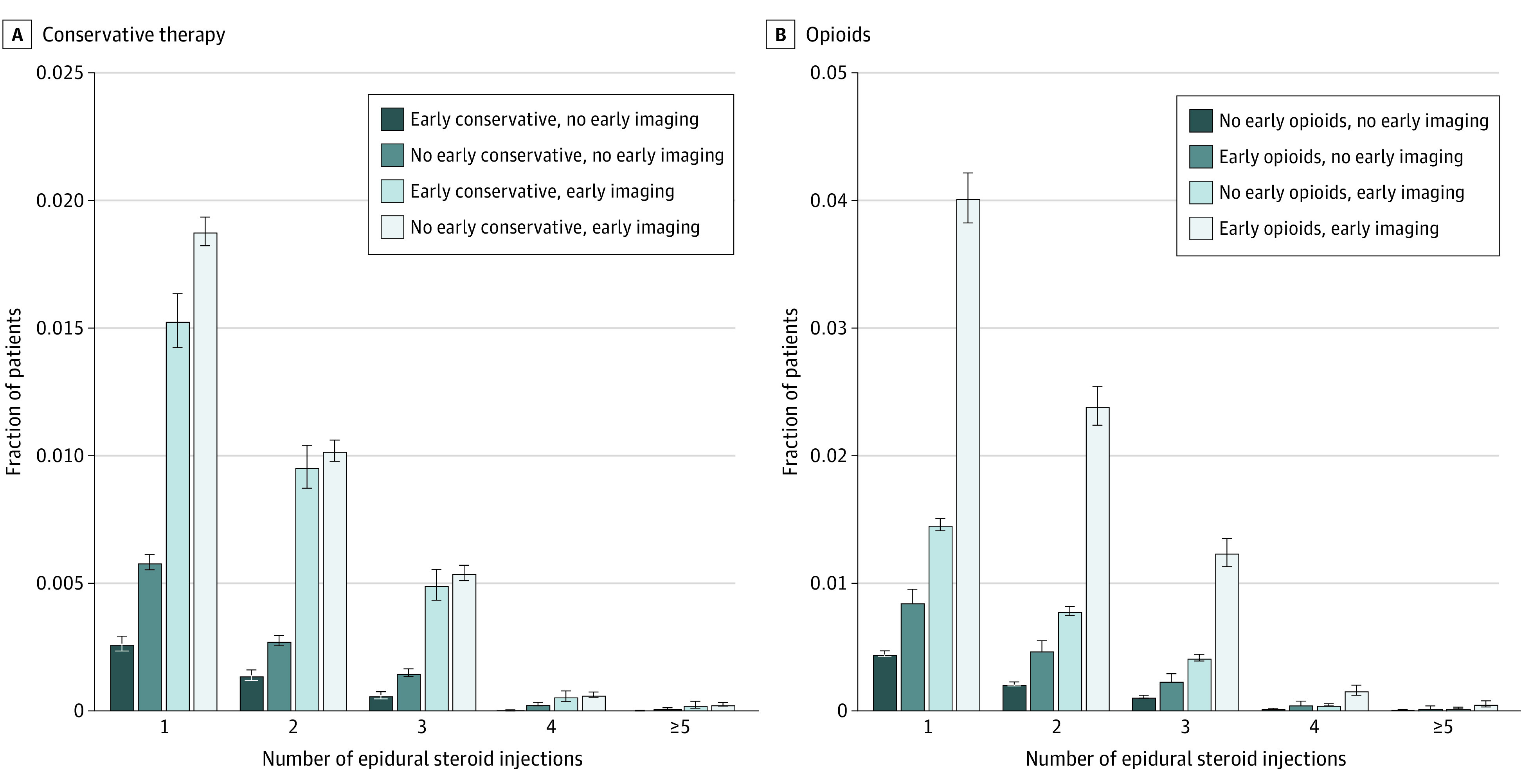
Use of Epidural Steroid Injections Stratified by Early Pain Management Graphs show differential epidural steroid injection use according to use of imaging and early conservative therapy (A) and opioids (B). Error bars indicate 95% CIs.

Patterns of pain-directed therapy received differed according to early clinical management approach. ESI was used more often among nonsurgical patients undergoing only early imaging than among patients receiving only early conservative therapy (5.95 cases per 100 patient-years vs 0.74 cases per patient-years). The prevalence of eventual ESI use was significantly higher among patients undergoing only imaging compared with those receiving only early conservative therapy (3.53% [95% CI, 3.46%-3.61%] vs 0.47% [95% CI, 0.43%-0.51%]; *P* < .001). Overall utilization of ESI during the year after neck pain onset was lowest for patients pursuing early conservative therapy (Figure 3A), no early opioids (Figure 3B), and no early imaging. Among patients eventually receiving an ESI, time to ESI was greatest among patients receiving only early conservative therapy, 131.4 days (95% CI, 123.22-139.57 days), vs 74.2 days (95% CI, 72.47-75.87 days) for those undergoing early imaging only and 72.7 days (95% CI, 69.07-76.39 days) for those receiving early conservative therapy and imaging) (both *P* < .001).

A total of 72 194 patients received opioids within 1 month of diagnosis. Among nonsurgical patients, 69 013 (10.3%) received opioids within 1 month of diagnosis. Long-term abstinence from opioids (ie, no opioids received during the year following initial diagnosis) was most common among nonsurgical patients receiving only early conservative therapy (81.3% vs 68.8% for those undergoing early imaging only and 73.8% for those receiving early conservative therapy and imaging). Even among patients who did eventually receive opioids, patients receiving only early conservative therapy had a later start of opioid use (157.4 days [95% CI, 156.0-158.9 days] vs 95.1 days [95% CI, 94.3-96.0 days] for those undergoing early imaging only and 128.9 days [95% CI, 126.8-131.0 days] for those receiving early conservative therapy and early imaging; both *P* < .001) and lower opioid burden (46.7 prescribed-days [95% CI, 44.5-48.9 prescribed-days] vs 59.8 prescribed-days [95% CI, 58.3-61.3 prescribed-days] for those undergoing early imaging only and 55.4 prescribed-days [95% CI, 53.8-56.9 prescribed-days] for those receiving neither; both *P* < .001). Even after adjusting for age, sex, and comorbidities, the diagnosing physician’s or practitioner’s specialty was associated with differential opioid use (eFigure 2 in the Supplement); notably, compared with those whose pain was diagnosed by family medicine physicians, patients who initiated care for neck pain with a pain medicine specialist were prescribed opioids for significantly longer (β = 23.7 prescribed-days; 95% CI, 18.9-28.4 prescribed-days).

## Discussion

Health care costs for spinal disorders have increased substantially over the last several decades and are expected grow further as a greater percentage of the population ages.^31^ Despite the high prevalence of neck pain in the general population, there are no established guidelines for evaluation and treatment of this common condition. The findings of this cross-sectional analysis of the IBM Watson Health MarketScan administrative claims database demonstrate that new-onset neck pain affects a diverse patient population with wide variation in treatment approaches. Slightly more than 1% of patients in this cohort underwent surgical therapy within the year after diagnosis.

Most health care spending occurred in the first 6 months following diagnosis, suggesting our cohort inclusion and exclusion criteria were successful in capturing patients with acute episodes of neck pain. Nearly 90% of nonsurgical patients did not accumulate further health care costs after the initial 6 months after diagnosis, supporting that idiopathic acute neck pain is a mostly self-limited condition. Among surgical patients, 47.2% received conservative therapy and 20.4% received ESI. The current literature remains unclear regarding the role of imaging on the diagnostic workup of new-onset neck pain. In the present study, 16.8% of patients not undergoing subsequent surgery and without evidence of tumor, fracture, trauma, or myelopathy underwent either MRI or computed tomography. More than 80% of these patients underwent imaging early after diagnosis, frequently before attempting conservative therapy. Although MRI is recommended for patients undergoing surgical intervention and those with a clinical presentation concerning for an urgent diagnosis such as tumor, concerns exist regarding incidental findings on advanced imaging and their relevance in relation to symptom onset.^32,33^ A recent meta-analysis^34^ comparing cervical spine MRI between subjects with and without neck pain largely found no differences and suggested few actionable radiological features. Despite this, our study demonstrated substantial treatment intensification; patients who underwent early imaging used ESI and opioids more frequently and earlier than those who received early conservative therapy. Additionally, imaging costs contribute greatly to health care expenditures. We speculate that for patients undergoing imaging without early conservative treatment, imaging often does not guide evidence-based intervention and may be superfluous. In a prior study,^4^ we demonstrated similar findings in patents with newly diagnosed idiopathic lower back pain, where among nonsurgical patients receiving no additional treatment, 36.6% of total health care spending was attributable to imaging. These patients eventually accumulated nearly 2.5 times higher health care costs compared with those receiving conservative therapy.^4^

The present study shows that patients receiving early conservative therapy may encounter lower cumulative health care expenditures compared with those undergoing early imaging without receiving conservative therapy. Those receiving early conservative therapy had significantly lower rates of ESI procedures, and patients receiving conservative therapy before imaging and ESIs experienced lower health care costs and resource utilization. These findings support those of prior studies^35,36^ conducted on smaller regional cohorts suggesting that delayed and late PT may be associated with increased health care costs and increased utilization of other services with unproven effectiveness. Early conservative therapy was associated with reduced opioid use and a higher prevalence of opioid-free treatment courses. These results are concordant with prior studies with alternative definitions of early conservative therapy and emphasize the importance of prompt conservative therapy initiation regardless of other intervention.^37^ Although few nonsurgical patients received conservative therapy, those who did frequently pursued conservative therapy early after diagnosis. The dichotomy between early conservative therapy or no conservative therapy may affect downstream care delivery for neck pain management.

There are few studies exploring population-level conservative therapy use among patients with neck pain. In a survey-based approach, Dikkers et al^38^ identified various practitioner-related impediments toward referral of subjects with neck pain to manual therapy. The primary emphasis of that study was exploring physician hesitancy with manual therapy referrals. Such qualitative investigations offer insight into causal practitioner-related factors; however, survey-based approaches do not evaluate the quantitative effect of identified motivating factors on empirical health care utilization. Cross-sectional research using population-level data sets such as MarketScan allow investigations into actual conservative therapy application and subsequent patient outcomes, such as cumulative costs and opioid burden. Such studies have been conducted for other spinal disorders; past studies suggest patients with low back pain who saw a physical therapist at the first point of care subsequently encounter lower costs and opioid use long term.^39,40,41^ Most published reports examining health care utilization focus on low back pain with less emphasis on new-onset idiopathic neck pain.

Overuse of opioids remains common. A study of patients undergoing total knee replacement demonstrated that conservative therapy use before and after surgery was associated with lower risk of long-term opioid use.^42^ The present study shows the benefit of early conservative therapy on reducing costs and opioid use in patients with acute neck pain. Few patients in our cohort initiated opioid use after first attempting conservative pain control, suggesting that conservative therapy was largely effective in both surgical and nonsurgical patients. Comparatively, those pursuing early imaging without conservative therapy were at elevated risk of opioid use. Nonetheless, opioids retain value as salvage therapies for acute pain management and refractory pain not improved by conservative treatment. Although most patients pursuing early conservative therapy never receive subsequent opioid prescriptions, those who do remain opioid free for much longer than conservative therapy–naive patients. Patients receiving early conservative therapy who do receive opioids were prescribed opioids for shorter durations than those not receiving early conservative therapy, suggesting that the use of opioids by these patients may be primarily for acute pain flares rather than long-term pain management. Finally, our findings on the association of practitioner specialty with opioid use are congruent with those presented in Azad et al.^40^ In our study, patients who initiated health care management with a pain medicine specialist accumulated significantly higher opioid use, whereas those initiating care with same-day physical therapy or chiropractor visits tended to accumulate lowest opioid use.

### Limitations

Limitations of this study include those inherent to all retrospective studies. Although we used multivariable models and regression-adjusted measures to reduce the effect of confounding features, there are likely uncollected and unavailable covariates that also influences the outcomes collected in our study. Although our study uses a big data approach that offers a geographically diverse assessment of care practices and outcomes, a potential weakness is the possibility of miscoded or missing values (which cannot be augmented using clinical records). Our study only includes subjects captured by the MarketScan database, so uninsured patients and those covered under public programs such as Medicaid were not available for analysis. Granularity of the features included in our study was limited to those coded for by either *International Classification of Diseases, Ninth Revision*, *International Statistical Classification of Diseases and Related Health Problems, Tenth Revision*, or *Current Procedural Terminology* coding. Furthermore, although large data sets such as the one included in our study allow for increased statistical power, interpretation of significant *P *values should be accompanied by evaluation of absolute and relative differences in the summary statistics of interest.^43^

## Conclusions

In this cohort of patients with acute neck pain without urgent diagnoses, early imaging followed by no further interventions contributed more than $100 million annually to health care expenditures. Early conservative therapy was associated with reduced long-term health care costs and opioid use. Early advanced imaging was associated with high costs, even among patients not receiving subsequent surgery, PT, CMT, or ESI. Timing of imaging and conservative therapy were associated with differential long-term patterns of care, suggesting certain care sequences alter the efficiency of health care delivery.

## Notes

Supplement. **eTable 1.** Study Coding Scheme **eTable 2.** Gamma Regression (Log-Link) on 1 Year Post-Diagnosis Total Healthcare Costs **eFigure 1.** CONSORT Diagram **eFigure 2.** Marginal Opioid Prescribing Duration Associated With Initial Provider Specialty
