# Supplementary material for: Health Care Resource Utilization in Management of Opioid-Naive Patients With Newly Diagnosed Neck Pain
Source: JAMA Netw Open. 2022 Jul 13;5(7):e2222062. doi: 10.1001/jamanetworkopen.2022.22062 (PMC9280399; doi:10.1001/jamanetworkopen.2022.22062)
Supplement: Supplement. — eTable 1. Study Coding Scheme eTable 2. Gamma Regression (Log-Link) on 1 Year Post-Diagnosis Total Healthcare Costs eFigure 1. CONSORT Diagram eFigure 2. Marginal Opioid Prescribing Duration Associated With Initial Provider Specialty [file jamanetwopen-e2222062-s001.pdf]

Supplemental Online Content

Jin MC, Jensen M, Zhou Z, et al. Health care resource utilization in management of opioid-naive patients with newly diagnosed neck pain. *JAMA Netw Open*. 2022;5(7):e2222062. doi:10.1001/jamanetworkopen.2022.22062

eTable 1. Study Coding Scheme
eTable 2. Gamma Regression (Log-Link) on 1 Year Post-Diagnosis Total Healthcare Costs
eFigure 1. CONSORT Diagram
eFigure 2. Marginal Opioid Prescribing Duration Associated With Initial Provider Specialty

This supplemental material has been provided by the authors to give readers additional information about their work.

© 2022 Jin MC et al. *JAMA Network Open.*

eTable 1. Study Coding Scheme

Characteristic	Codes Used
Inclusion Diagnosis	
Neck Pain without Myelopathy	353.2, 739.1, 721.0, 722.0, 722.4, 722.91, 723.1, 723.2, 723.3, 723.4, 723.7, 723.8, 723.9, 805.11, 806.11, 847.0, G54.2, M47.22, M47.23, M47.812, M47.813, M47.892, M47.893, M54.12, M54.13, M54.2, M50.10, M50.11, M50.12, M50.120, M50.121, M50.122, M50.123, M50.13, M50.20, M50.21, M50.22, M50.220, M50.221, M50.222, M50.223, M50.23, M50.30, M50.31, M50.32, M50.320, M50.321, M50.322, M50.323, M50.33, M50.80, M50.81, M50.82, M50.820, M50.821, M50.822, M50.823, M50.83, M50.90, M50.91, M50.92, M50.920, M50.921, M50.922, M50.923, M50.93, M53.0, M53.1, M53.2X2, M53.2X3, M53.82, M53.83, M54.02, M54.03, M99.01, S13.4XXA, S13.8XXA
Exclusion Diagnoses	
Traumatic Cervical Disc Dislocation	839.00, 839.01, 839.02, 839.03, 839.04, 839.05, 839.06, 839.07, 839.08, 839.10, 839.11, 839.12, 839.13, 839.14, 839.15, 839.16, 839.17, 839.18
Cervical Fracture	805.00, 805.01, 805.02, 805.03, 805.04, 805.05, 805.06, 805.07, 805.08, 805.10, 805.11, 805.12, 805.13, 805.14, 805.15, 805.16, 805.17, 805.18
Malignancy	140, 141, 142, 143, 144, 145, 146, 147, 148, 149, 150, 151, 152, 153, 154, 155, 156, 157, 158, 159, 160, 161, 162, 163, 164, 165, 166, 167, 168, 169, 170, 171, 172, 173, 174, 175, 176, 177, 178, 179, 180, 181, 182, 183, 184, 185, 186, 187, 188, 189, 190, 191, 192, 193, 194, 195, 196, 197, 198, 199, 200, 201, 202, 203, 204, 205, 206, 207, 208, 209, 210, 211, 212, 213, 214, 215, 216, 217, 218, 219, 220, 221, 222, 223, 224, 225, 226, 227, 228, 229, 230, 231, 232, 233, 234, 235, 236, 237, 238, 239
Other	038, 334.1, 338.2, 342, 343, 720, 739.1, 787.6, 788.3, 995.9
Procedures	
Surgery	
Anterior Decompression	22856, 22861, 22864, 0095T, 63075, 22859, 63081
Anterior Fusion	22548, 22554, 22551, 22845, 22843, 22853, 22854
Posterior Decompression	63001, 63015, 63020, 63040, 63045, 63050, 63051, 22210
Posterior Fusion	22590, 22595, 22600, 22840, 22842, 22843
Imaging	
X-Ray	72040, 72050, 72052
CT	72125, 72126, 72127
MRI	72141, 72156, 72142
Non-Surgical Treatments	
ESI	62310, 64479, 64480
Chiropractic Manipulative Therapy	98940, 98941, 98942, 98943
Physical Therapy	97010, 97014, 97001, 97002, 97161, 97162, 97163, 97164, 97140, 97110, 97012

© 2022 Jin MC et al. *JAMA Network Open.*

eTable 2. Gamma Regression (Log-Link) on 1 Year Post-Diagnosis Total Healthcare Costs			
**Characteristics**	e^{β}	95% CI	*p*-value
**Received Surgery**	27.295	25.549 to 29.159	<0.001
**Early Imaging**			
None (ref)			
X-Ray	1.105	1.086 to 1.125	<0.001
Advanced Imaging	2.691	2.624 to 2.759	<0.001
X-Ray and Advanced Imaging	3.517	3.381 to 3.658	<0.001
**Early Opioids**	1.408	1.374 to 1.443	<0.001
No (ref)			
Yes			
**Early Conservative**	0.752	0.738 to 0.765	<0.001
No (ref)			
Yes			
**Age (years, continuous)**	0.997	0.996 to 0.998	<0.001
**Sex**			
Male (ref)			
Female	1.004	0.989 to 1.020	0.589
**Comorbidities**			
Congestive Heart Failure	0.884	0.817 to 0.957	0.002
Cardiac Arrhythmia	0.974	0.933 to 1.016	0.217
Valvular Disease	0.968	0.917 to 1.022	0.235
Pulmonary Circulation Disorders	0.871	0.761 to 0.997	0.041
Peripheral Vascular Disorders	0.876	0.823 to 0.933	<0.001
Hypertension Uncomplicated	0.989	0.969 to 1.011	0.318
Hypertension Complicated	0.966	0.900 to 1.038	0.337
Paralysis	1.299	1.008 to 1.675	0.039
Other Neurological Disorders	1.121	1.061 to 1.184	<0.001
Chronic Pulmonary Disease	0.990	0.961 to 1.021	0.521
Diabetes Uncomplicated	1.031	0.999 to 1.064	0.057
Diabetes Complicated	0.969	0.910 to 1.033	0.325
Hypothyroidism	1.007	0.977 to 1.038	0.665
Renal Failure	0.850	0.779 to 0.927	<0.001
Liver Disease	1.162	1.088 to 1.241	<0.001
Peptic Ulcer Disease excluding bleeding	1.117	0.954 to 1.308	0.162
AIDS/HIV	1.147	0.944 to 1.395	0.160
Rheumatoid Arthritis/collagen	1.032	0.973 to 1.094	0.280
Coagulopathy	0.954	0.847 to 1.076	0.435
Obesity	1.072	1.030 to 1.115	<0.001
Weight Loss	0.945	0.866 to 1.032	0.197
Fluid and Electrolyte Disorders	0.950	0.896 to 1.007	0.077
Blood Loss Anemia	0.859	0.715 to 1.032	0.098
Deficiency Anemia	1.017	0.957 to 1.081	0.579
Alcohol Abuse	0.990	0.904 to 1.085	0.832
Drug Abuse	1.046	0.941 to 1.163	0.395
Psychoses	0.837	0.748 to 0.936	0.001
Depression	1.101	1.071 to 1.132	<0.001

© 2022 Jin MC et al. *JAMA Network Open.*

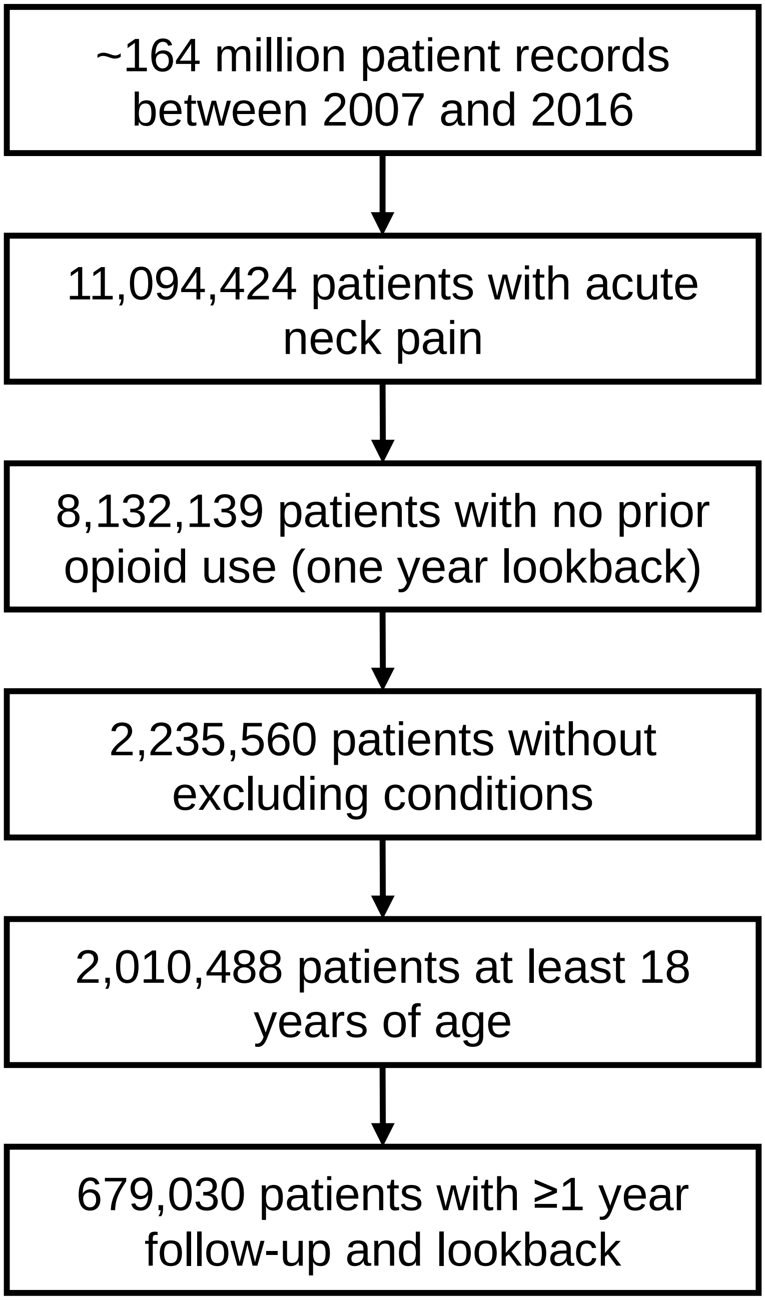

eFigure 1. CONSORT Diagram

© 2022 Jin MC et al. *JAMA Network Open.*

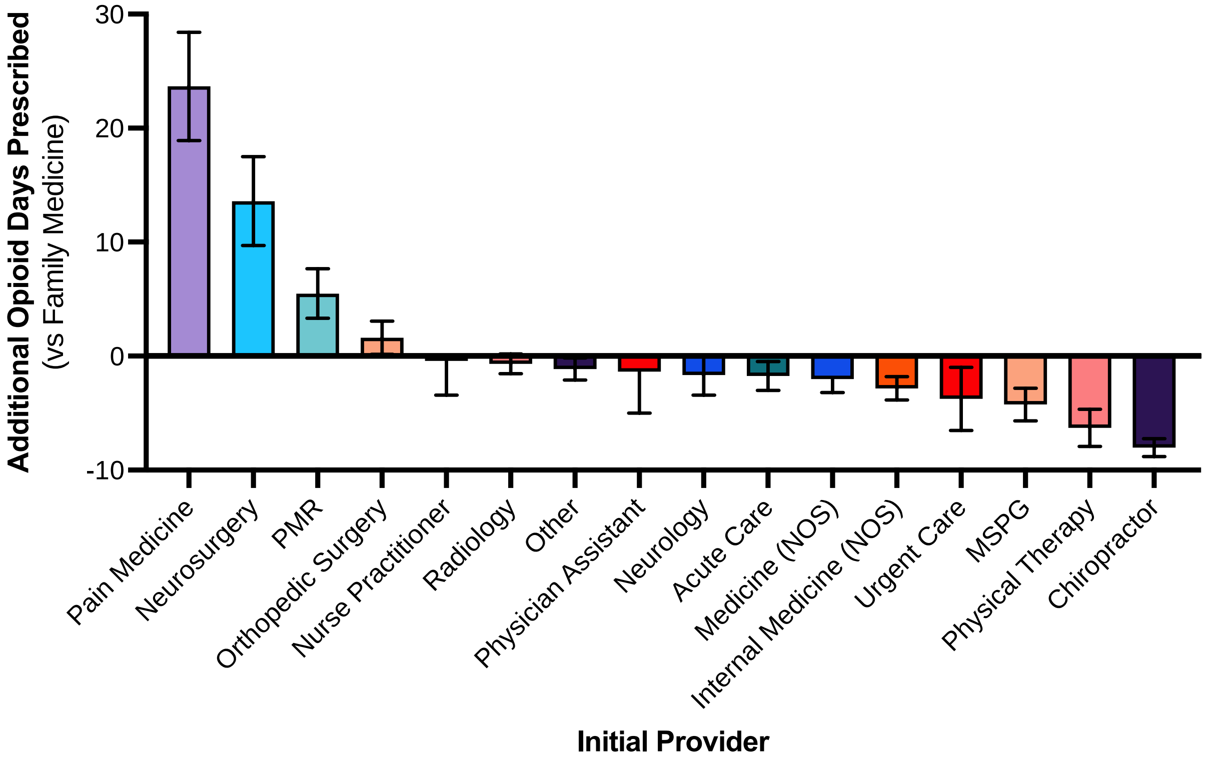

eFigure 2. Marginal Opioid Prescribing Duration Associated With Initial Provider Specialty. Regression-adjusted prescribed opioid duration stratified by initial provider type after diagnosis. Error bars indicate 95% confidence intervals.

© 2022 Jin MC et al. *JAMA Network Open.*
